# On-Line Detection and Segmentation of Sports Motions Using a Wearable Sensor †

**DOI:** 10.3390/s18030913

**Published:** 2018-03-19

**Authors:** Woosuk Kim, Myunggyu Kim

**Affiliations:** Creative Content Research Division, Electronics and Telecommunications Research Institute, 218 Gajeong-ro, Yuseong-gu, Daejeon 34129, Korea; mgkim@etri.re.kr

**Keywords:** sports motion, detection, segmentation, wearable sensor, deep neural networks

## Abstract

In sports motion analysis, observation is a prerequisite for understanding the quality of motions. This paper introduces a novel approach to detect and segment sports motions using a wearable sensor for supporting systematic observation. The main goal is, for convenient analysis, to automatically provide motion data, which are temporally classified according to the phase definition. For explicit segmentation, a motion model is defined as a sequence of sub-motions with boundary states. A sequence classifier based on deep neural networks is designed to detect sports motions from continuous sensor inputs. The evaluation on two types of motions (soccer kicking and two-handed ball throwing) verifies that the proposed method is successful for the accurate detection and segmentation of sports motions. By developing a sports motion analysis system using the motion model and the sequence classifier, we show that the proposed method is useful for observation of sports motions by automatically providing relevant motion data for analysis.

## 1. Introduction

Analyzing quality of motion is essential for evaluating the performance of athlete’s movements in sports [1]. The analysis begins by observation; sports coaches or teachers need to know how movements are carried out before judging quality. In the sports science literature, systematic models have been introduced as aids to observation [2,3]. For example, phase analysis refers to dividing up a movement into relevant sub-parts; temporal analysis means finding out temporal relationships (timing or rhythm) of movements; and, critical features define elements of a movement, which decide performance of a skill [2]. Although terms and definitions may vary, it is common that a motion is perceived as a sequential pattern of movements and observation is thought of as a task to inspect spatio-temporal characteristics of the pattern in analysis of sports motions.

Technologies have already been adopted for helping observation of sports motions [4]. Using video cameras for recording motions of athletes is a simple but efficient way to review their techniques or skills, coaches and athletes can examine their movements frame-by-frame or archive them for future comparison. Moreover, recent advances in sensor hardware and information technologies show huge potential for further improvements. Image sensors with high resolution, inertial measurement units (IMUs) with great accuracy, or depth cameras make it possible to provide detailed information about motions, which cannot be obtained by human eyes easily. In addition, gesture recognition or activity detection using machine learning makes it possible to automate observation processes.

Video-based gesture and action recognition has always been of great concern for many researchers. With the successes in image classification based on deep learning, deep neural networks also seem to be promising for gesture and action recognition as well. Ji, et al. [5] proposed a three-dimensional (3D) convolutional neural networks (CNNs) [6] for human activity recognition, Molchanov, et al. [7] combined recurrent neural networks (RNNs) [8] and CNNs for gesture recognition, and Simonya and Zisserman [9] shown dual architecture with temporal and spatial CNNs for action recognition. Lea, et al. [10] tried to segment the daily actions using temporal CNNs, but not for sports motions. Hidden markov models (HMMs) [11] have been known to be effective for segmentation due to innate temporal properties. Li et al. [12] introduced detection and segmentation of sports actions into phases using HMMs, however the method was applied to off-line videos thus not for real-time uses.

Depth cameras can provide useful information about human movements represented in 3D space [13]. Zhang et al. [14] used a depth camera to recognize and segment golf swings for grading motion quality. Gong et al. [15] defined an approach to recognize the temporal structure of actions. Wu, et al. [16] jointly utilized RGB and depth images in a hybrid recognition model using HMMs and deep belief networks. Although depth cameras have advantages in providing skeletal motions of a human body, their low framerate is not suitable for highly dynamic sports motions.

Wearable inertial sensors are suitable for recognition of actions [17] and for analysis of sports motions [18] as well, since they have less constraints on time and space for acquiring motion data. Mannini and Sabatini [19] proposed a method for recognizing gait motions using HMMs, Saeedi, et al. [20] introduced HMM-based framework for the segmentation of motions, and Weber, et al. [21] used long short-term memory (LSTM) [22] for early recognition of actions. Although these works include, explicitly or implicitly, separation of motions from other types, segmentation of a single action into sub-phases is not considered. The work of Yang, et al. [23] is conceptually similar to ours; they provided a method to segment tennis serves into relevant phases for quantitative analysis. Yet, it does not find exact boundaries between phases and can only be applied to tennis serves.

In this paper, we propose a novel approach to detect and segment sports motions using a wearable sensor. Our main concern is to support observation of sports motions in analysis tasks by automatically classifying states (or phases) of the recognized motions. The automatic classification is processed on-line from continuous sensor inputs, thus users can get feedback immediately. For explicit segmentation of a motion into phases, a motion model is defined as a sequence of sub-motions with boundary states. A sequence classifier that is based on deep neural networks is designed to detect sports motions from continuous sensor inputs. For evaluation, two types of motions (soccer kicking and two-handed ball throwing) were collected. The evaluation was carried out with two questions in mind: how well the proposed method detects an occurrence of a motion to be analyzed and how accurately the detected motion is segmented into the pre-defined states. In addition, a sports motion analysis system [24] based on the proposed method is presented to demonstrate the applicability in real-world situations.

## 2. Materials and Methods

### 2.1. Motion Model

As stated in the introduction, we perceive a sports motion as a sequence of sub-motions or phases. It is similar to state transition models, like left-right models, in HMM-based gesture recognition methods [25]. Although segmentation is important for recognition accuracy [17,26], finding exact boundaries of states is not the main concern of conventional gesture recognition. For explicit segmentation, we further divide a sub-motion $M_{sub}$ into a sequence of three states as

(1) $$M_{sub}=\left(m_{s},\;m_{p},\;m_{e}\right),$$

where $m_{s}$, $m_{p}$, and $m_{e}$ are *start*, *performing*, and *end* states, respectively.

**Figure 1:**
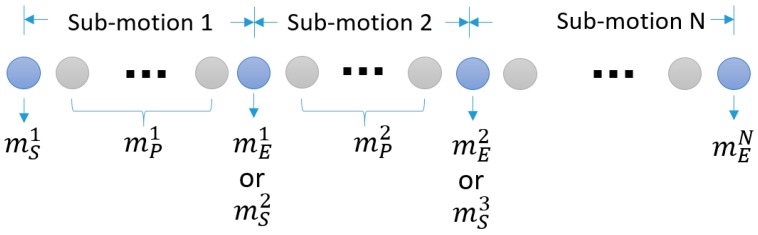
A motion model with N sub-motions represented as a sequence of states and its correspondence to sensor inputs (a circle represent a sensor input sample).

The start and end states imply boundaries of a sub-motion and the performing state is considered as relevant movement. It is assumed that transition between sub-motions is instant so the length of a boundary state is one (each boundary state corresponds to only a single input sample) and the end state of a sub-motion coincides with the start state of the next sub-motion. Consequently, a sports motion M is represented as a sequence of states:

(2) $$M=\left(m_{s}^{1},\;m_{p}^{1},\;m_{e}^{1}\;or\;m_{s}^{2},m_{p}^{2},\;\ldots ,\;m_{e}^{N-1}\;or\;m_{s}^{N},\;m_{p}^{N},\;m_{e}^{N}\right),$$

in which sub-motions are overlapped at boundaries (Figure 1). 

### 2.2. On-Line Detection and Segmentation

Detection of a motion from continuous sensor inputs is described in a two-step process: (1) motion states corresponding to an input sequence is estimated, and (2) the state pattern of the motion is searched from the sequence of the estimated states.

To estimate states, a sequence classifier N is defined as:

(3) N : x↦y,

(4) $$x=\left(x_{1},\;x_{2},\;\ldots ,\;x_{L}\right),$$

(5) $$y=\left(y_{1},\;y_{2},\;\ldots ,\;y_{L}\right),$$

and

(6) $$y_{l}=\left(y_{l}^{0},\;y_{l}^{1},\;\ldots ,\;y_{l}^{k}\right),$$

where x is a sequence of L feature vectors and y is a sequence of state probabilities. The state probability y is encoded as a vector, where $y_{l}^{k}$ is the probability of being state *k* at the *l*-th input sample (0≤k≤N and *k* = 0 is the none or unknown state [27]). In the following section, we will describe the implementation details of the sequence classifier based on deep neural networks. 

Because of temporal variances of motions, especially large in sports motions, it is not guaranteed that a whole motion is contained in the input sequence of a fixed length L. Increasing the size of an input to the classifier is not a feasible option due to the computational cost and not preferable for real-time operation. Instead, the outputs of the classifier are accumulated to build a longer sequence of state probabilities. Let $y_{s}^{t}$ be state probabilities of the input sample at time s (s>0) estimated at time t (t>0), then the accumulated state sequence at time t is represented as:

(7) $$a(t)=\left(\ldots ,\;y_{t-L-2}^{t-3},\;y_{t-L-1}^{t-2}\;,\;y_{t-L}^{t-1}\;,\;y_{t-L+1}^{t},\;\ldots ,\;y_{t}^{t}\right)$$

From the accumulated sequence of state probabilities, a motion is detected by searching the pattern of states defined by a motion model. In our implementation, the state pattern (or only boundary states for simplicity) is searched from the end (the most recent state) of the accumulated sequence in reverse direction. The longest common subsequence (LCSS) algorithms [28] can also be used, since it is a similar to string matching problem if we substitute states for characters.

As we explicitly defined state boundaries in the motion model, it is straightforward to segment the detected motions. From the accumulated sequence of state probabilities (Equation (6)), temporal indices of boundary states can be found by:

(8) $$b_{k^{\prime }}=\underset{l}{\mathrm{argmax}\;}y_{l}^{k^{\prime }},\;\mathrm{if}\;\mathrm{and}\;\mathrm{only}\;\mathrm{if}\;b_{k_{1}^{\prime }}<b_{k_{2}^{\prime }}\;for\;k_{1}^{\prime }<k_{2}^{\prime }$$

where $k^{\prime }$ indicates indices of boundary states.

### 2.3. Implemetation

#### 2.3.1. Hardware for Motion Data Acquisition

**Figure 2:**
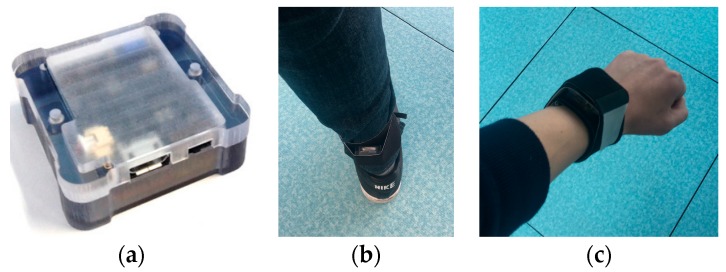
The wearable sensor used for recording motion data: (**a**) the appearance of the sensor; (**b**) the sensor worn on the ankle; (**c**) the sensor worn on the wrist.

To gather motion data for training and evaluation, we used a commercially available wearable sensor [29]. The sensor consists of a tri-axis accelerometer (±16 g), a tri-axis gyroscope (±2000°/s), and a tri-axis magnetometer with a 2.4 GHz wireless communication module (Figure 2a). For recording, it was configured to output acceleration readings in the global (earth) coordinate system (static error < 0.5° and dynamic error < 1.5° in orientation estimation) and gyroscope readings (angular velocity) in the sensor’s local coordinate system at a rate of 100 Hz.

**Figure 3:**
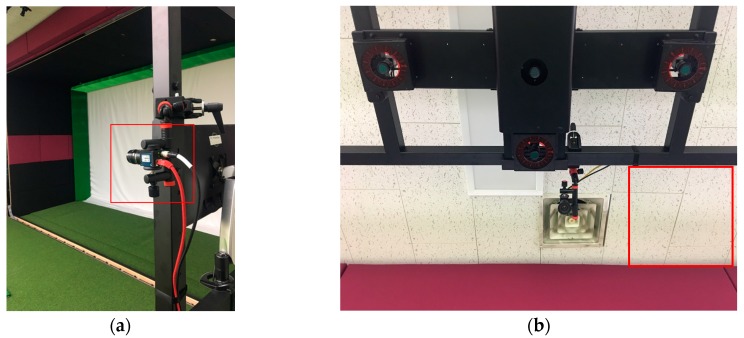
The cameras used for capturing images along with the wearable sensor: (**a**) the side view camera; and, (**b**) the top view camera.

At the same time, two high speed cameras (640 × 480@100 fps, CREVIS Co., Ltd., Yongin-si, Korea) were used to the record images of a motion viewed from the side and top (Figure 3). Capturing images from the cameras was synchronized with the wearable sensor, so it was possible to find out temporal correspondence between images and sensor data. The temporal mapping between images and sensor data were used for labeling.

#### 2.3.2. Datasets

Two types of motion data were gathered for training and evaluation: soccer kicking and two-handed ball throwing. When collecting, approximately five seconds of data (from the sensor and cameras) were recorded for a single performance. As the lengths of the two types of motions were usually shorter than five seconds, recorded data may include irrelevant motions like walking or stepping.

A total of 404 soccer kicking motions were recorded with the wearable sensor being attached to the behind of a kicking leg’s ankle (Figure 2b). The motion model of soccer kicking was defined as a sequence of five phases [30,31] with six boundary states as in Table 1. The recorded motions were labeled according the state definition and irrelevant parts were marked as none or unknown states.

For two-handed ball throwing, 333 motions were recorded with the sensor on the wrist (Figure 2c). The motion model was defined as a sequence of three phases with four boundary states as in Table 2.

#### 2.3.3. Sequence Classifier N

**Figure 4:**
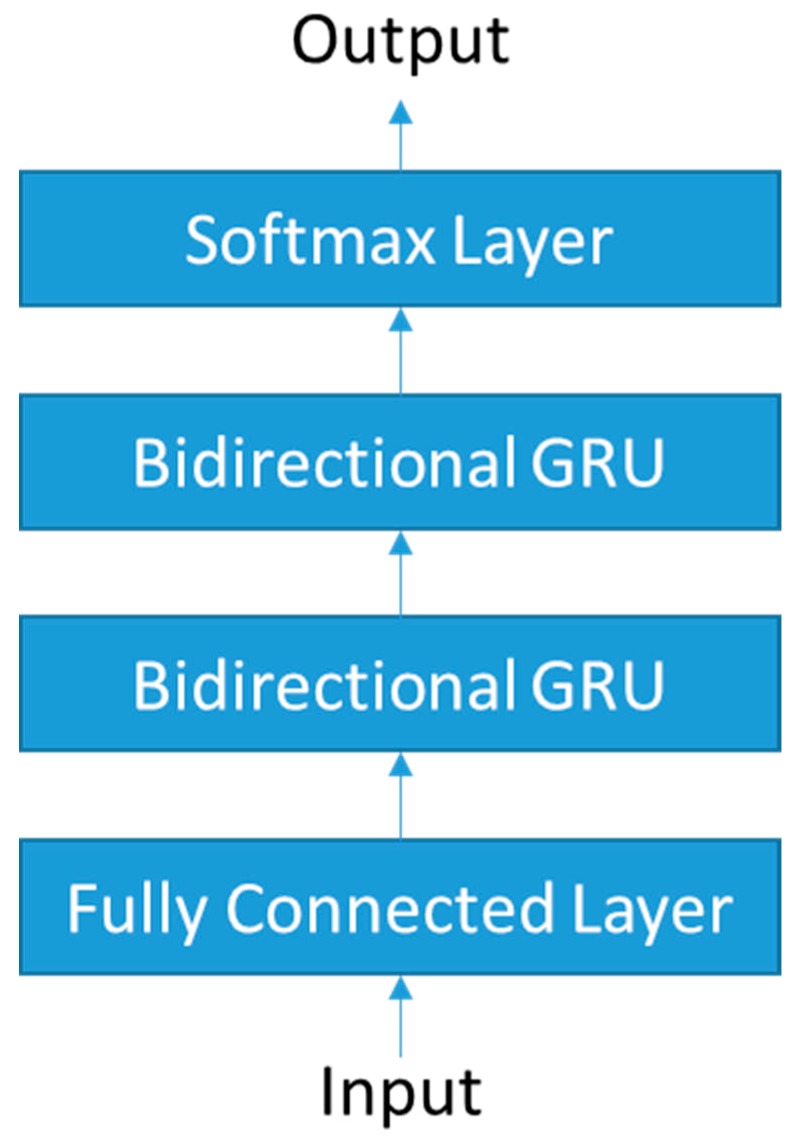
The deep neural network model of the sequence classifier.

The sequence classifier N is defined based on deep neural networks. Specifically, bidirectional recurrent neural networks (bidirectional RNNs) [32] are used because of effectiveness in sequence labeling tasks [27]. Figure 4 shows the architecture of the network.

The input takes a sequence of 100 feature vectors (L=100) created from the sensor data. The feature vector consists of 11 elements as follows:

- Acceleration along *Z*-axis (opposite direction of gravity)
- Magnitude of acceleration
- Magnitude of angular velocity
- Magnitude of the first derivative of acceleration
- Magnitude of the first derivative of angular velocity
- Magnitude of the second derivative of acceleration
- Magnitude of the second derivative of angular velocity
- Angular difference between adjacent acceleration vectors
- Angular difference between adjacent angular velocity vectors
- Angular difference between adjacent vectors of the first derivative of acceleration
- Angular difference between adjacent vectors of the first derivative of angular velocity

The first hidden layer is a fully connected layer with a size of 48, which uses exponential linear units (ELUs) [33] as an activation function. For the bidirectional recurrent layer, gated recurrent units (GRUs) [34] are used instead of LSTM because GRUs have less parameters (less computational cost) but similar performance when compared to LSTM. The two bidirectional GRU layers are stacked on the first hidden layer and the cell size of each is 48 and 32, respectively. The last layer is a softmax layer, which outputs a sequence of state probability vectors. The sizes of the probability vectors are twelve and eight (both include additional none states) for soccer kicking and two-handed ball throwing, respectively. Batch normalization [35] is applied to all the hidden layers and dropout [36] is used except for the output layer. The network was implemented using Keras [37] with theano backend [38].

#### 2.3.4. Training

From the datasets, 80% of the recorded motions (324 for soccer kicking and 267 for two-handed ball throwing) were randomly selected for training. Since the input size of the classifier N is fixed to 100, we further sliced the recorded motions. Using the sliding window method, 126,852 and 95,145 sequences of 100 feature vectors were extracted, respectively, from the soccer kicking and two-handed throwing motions.

For the cost function, weighted categorical cross-entropy was used:

(9) $$ℒ\left(y,\;\hat{y}\right)=-\sum_{i}\alpha_{i}y_{i}log\hat{y_{i}},$$

where $\alpha_{i}$ is a weight inversely proportional to the total number of state i in datasets. As the numbers of labels is statistically unbalanced, classification errors related to the labels of small numbers would be ignored easily. The weight can add significance on errors in boundary states, which are important for segmentation but much fewer than the others.

The classification networks for soccer kicking and two-handed ball throwing were trained using Adam optimizer [39] with a batch size of 100 for 30 and 20 epochs, respectively.

## 3. Results

### 3.1. Evaluation

Detection and segmentation accuracy of the proposed method was evaluated using the trained networks in the previous section. As test sets, 20% from the datasets, excluding training sets, were used for evaluation (80 for soccer kicking and 66 for two-handed ball throwing). Each motion sample from the test sets was separately fed into the detection and segmentation process as if it were an on-line data stream. Only for successfully detected samples, segmentation errors were measured by comparing the temporal indices of boundary states between the estimated state sequences and the manually labeled data.

For soccer kicking, 76 out of 80 samples were successfully detected. Table 3 shows the segmentation errors measured in frames (one frame is 10 ms) of soccer kicking motions.

For two-handed throwing, 62 out of 66 samples were successfully detected. Table 4 shows the segmentation errors of two-handed ball throwing motions.

Except the start and end of motions ($L_{S}^{1},{\;L}_{S}^{11},{\;L}_{T}^{1}$, and $L_{T}^{7})$, segmentation errors were less than three frames (30 ms). When considering difficulties in discrimination of adjacent images when labeling motion data, the result proves that the proposed method can segment motions into phases very well.

From inspection of the recorded data and labels, we found that the reason for relatively large errors of the start and end states is due to labeling errors. For example, some participants stayed still while the others swung their arms back and forth at the end of throwing ($L_{T}^{7})$ or a few of participants jumped at the end of kicking ($L_{S}^{11})$. These inconsistencies in the execution of movements by people made it difficult to determine boundaries by human perception. Also, for landing of a kicking leg ($L_{S}^{1})$, the motion was less dynamic (several consecutive images did not visually change much), so the boundaries were ambiguous. Hence, ways to overcome errors caused by manual labeling are required for the further improvement of accuracy of the proposed method.

### 3.2. Sports Motion Analysis System

**Figure 5:**
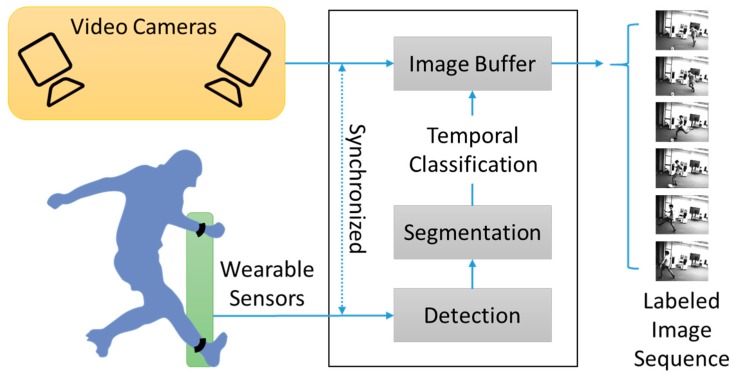
The conceptual structure of the sports motion analysis system.

Based on the proposed method, a sports motion analysis system was developed [24]. Figure 5 shows the conceptual structure of the system. The system uses wearable sensors and cameras to capture user motions. Acquisition of images and sensor data is synchronized, so it is possible to find out temporal mappings between them. When a user performs a sports motion to be analyzed, the system automatically detects and segments the motion according to the method that is described in Section 2.2. Using segmentation results and temporal mappings, the system classifies images from the cameras and provides the labeled images for analysis.

**Figure 6:**
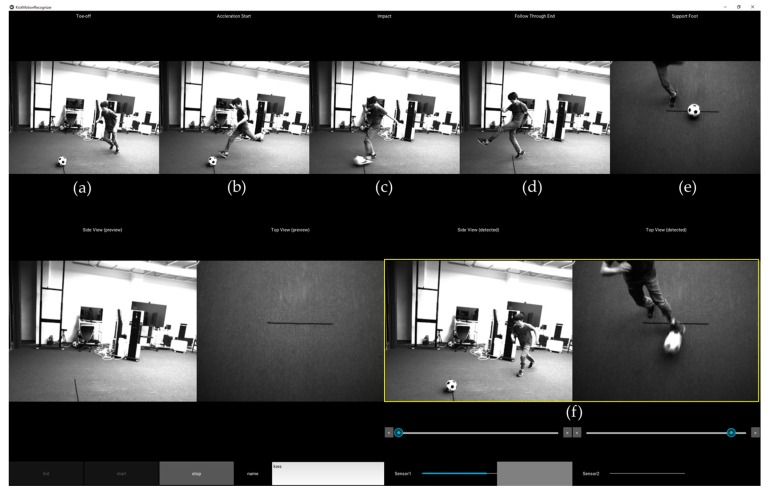
An example screenshot of soccer kicking analysis: (**a**) toe-off ($L_{S}^{3}$); (**b**) maximum hip extension ($L_{S}^{5}$); (**c**) ball impact ($L_{S}^{7}$); (**d**) end of kicking ($L_{S}^{11}$); (**e**) top view image for checking ball-foot relationship; and, (**f**) detected motion sequences for review.

**Figure 7:**
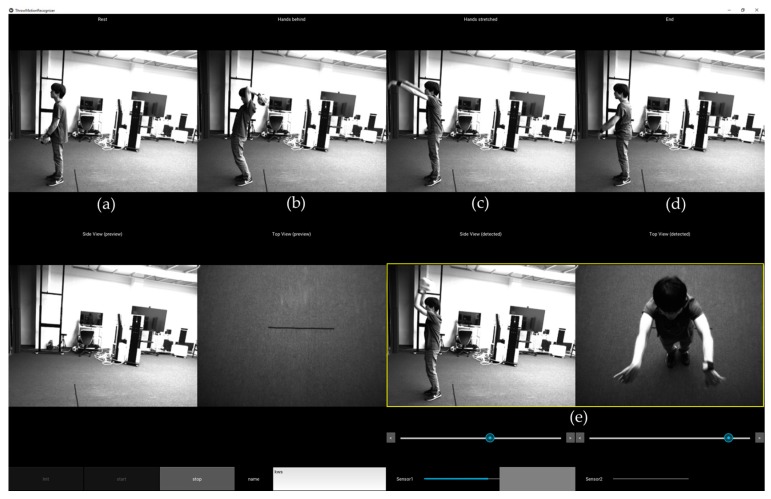
An example screenshot of two-handed ball throwing analysis: (**a**) ready ($L_{T}^{1})$; (**b**) two hands behind of a head ($L_{T}^{3})$; (**c**) maximum arm stretch ($L_{T}^{5})$; (**d**) end of throwing ($L_{T}^{7})$; and, (**e**) detected motion sequences for review.

The system was implemented and tested with the motion models and the trained classifiers for soccer kicking and two-handed ball throwing. For soccer kicking, it was designed to obtain five still images of interest, which include four side view images: toe-off ($L_{S}^{3}$), maximum hip extension ($L_{S}^{5}$), ball impact ($L_{S}^{7}$), and end of kicking ($L_{S}^{11}$), as shown in Figure 6. In addition, a top view image is provided for checking the spatial relationship between a support leg and a ball. Similarly, Figure 7 shows four side view images of the detected two-handed ball throwing motion: ready ($L_{T}^{1})$, two hands behind of a head ($L_{T}^{3})$, maximum arm stretch ($L_{T}^{5})$, and end of throwing ($L_{T}^{7})$. Also, image sequences of the detect motion are presented for frame-by-frame inspection and are archived for future analysis.

The system was tested by a small number of participants, including former student athletes. Rather than quantitative evaluation, we tried to focus on observing usability as a motion analysis tool. During hours of testing, we have found that the participants were able to check their postures and movements easily and compare their performances to the others. Although we were not able to evaluate the system quantitatively in a full scale, the results from the test shown the applicability of the proposed method in real-world situations.

## 4. Discussion and Conclusions

In this paper, we presented a method to detect and segment sports motions using a wearable sensor. A sequence classifier based on bidirectional RNNs and a motion model with explicit boundary states were defined for the detection and segmentation from continuous sensor inputs. The evaluation on datasets of two types (soccer kicking and two-handed ball throwing) shown that the proposed method was successful at detecting and segmenting motions to be analyzed. Also, the sports motion analysis system based on the proposed method was proved to be helpful for sports motion analysis through the tests in real-world conditions.

For some motion states (mostly the start and end of a whole motion), segmentation errors were relatively larger than the others. By inspecting datasets and labels, we found that it is mainly caused by either the inconsistency of movements (of the same state) or ambiguity in choosing boundaries between movements with little dynamics (slowly changing motion). So, it will be our next goal to find out ways to detect and segment motions robustly, regardless of irregular movements and labeling errors.

In addition, there seem to be alternative approaches, although they are from different domains, which can be applied to motion segmentation. For example, attention [40] is used for temporally aligning speeches and sentences, and connectionist temporal classification (CTC) [26,27] is proposed for implicitly segmenting speeches or hand writings. Adopting these methods for improving motion segmentation and comparing with the current work will be interesting future work.

## Figures and Tables

**Table 1:** The motion model for soccer kicking.

State	Description	Label
$m_{s}^{1}$	Landing of a kicking leg	$L_{S}^{1}$
$m_{p}^{1}$	Last step of a kicking leg before impact	$L_{S}^{2}$
$m_{e}^{1}$ or $m_{s}^{2}$	Toe-off of a kicking leg	$L_{S}^{3}$
$m_{p}^{2}$	Backswing of a kicking leg	$L_{S}^{4}$
$m_{e}^{2}$ *or* $m_{s}^{3}$	Maximum hip extension	$L_{S}^{5}$
$m_{p}^{3}$	Acceleration of a kicking leg	$L_{S}^{6}$
$m_{e}^{3}$ *or* $m_{s}^{4}$	Ball impact	$L_{S}^{7}$
$m_{p}^{4}$	Follow-through	$L_{S}^{8}$
$m_{e}^{4}$ or $m_{s}^{5}$	Toe speed inflection	$L_{S}^{9}$
$m_{p}^{5}$	Landing of a kicking leg	$L_{S}^{10}$
$m_{e}^{5}$	End of kicking	$L_{S}^{11}$

**Table 2:** The motion model for two-handed ball throwing.

State	Description	Label
$m_{s}^{1}$	Ready	$L_{T}^{1}$
$m_{p}^{1}$	Brining two hands behind	$L_{T}^{2}$
$m_{e}^{1}$ or $m_{s}^{2}$	Two hands behind of a head	$L_{T}^{3}$
$m_{p}^{2}$	Arms foward	$L_{T}^{4}$
$m_{e}^{2}$ or $m_{s}^{3}$	Maximum arm stretch	$L_{T}^{5}$
$m_{p}^{3}$	Follow-through	$L_{T}^{6}$
$m_{e}^{3}$	End of throwing	$L_{T}^{7}$

**Table 3:** The average errors on segmentation for soccer kicking.

State	Avg. Segmentation Errors(in Frames)
Landing of a kicking leg ($L_{S}^{1})$	8.17
Toe-off of a kicking leg ($L_{S}^{3})$	2.82
Maximum hip extension ($L_{S}^{5})$	2.092
Ball impact ($L_{S}^{7})$	0.723
Toe speed inflection ($L_{S}^{9})$	2.855
End of kicking ($L_{S}^{11})$	5.342

**Table 4:** The average errors on segmentation for two-handed ball throwing.

State	Avg. Segmentation Errors(in Frames)
Ready ($L_{T}^{1})$	4.032
Two hands behind of a head ($L_{T}^{3})$	1.564
Maximum arm stretch ($L_{T}^{5})$	1.419
End of throwing ($L_{T}^{7})$	24.11
